# Depression, Social Support, and Coping Styles among Pregnant Women after the Lushan Earthquake in Ya’an, China

**DOI:** 10.1371/journal.pone.0135809

**Published:** 2015-08-13

**Authors:** Jianhua Ren, Xiaolian Jiang, Jianrong Yao, Xirong Li, Xinghui Liu, Meiche Pang, Chung Lim Vico Chiang

**Affiliations:** 1 Department of Obstetrics, West China Second University Hospital, Chengdu, Sichuan, China; 2 West China School of Nursing/West China Hospital, Sichuan University, Chengdu, Sichuan, China; 3 School of Nursing, The Hong Kong Polytechnic University, Hong Kong, Hong Kong; 4 Obstetrics Department, Ya’an People’s Hospital, Ya’an, Sichuan, China; University of Rennes-1, FRANCE

## Abstract

**Aim:**

The aim of this study is to assess the depression of pregnant women in the aftermath of an earthquake, and to identify the social support that they obtained, their coping styles and socio-demographic factors associated with depression.

**Methods:**

A total of 128 pregnant women from three hospitals in the epicenter area were recruited immediately after the Ya’an earthquake. Their depression was investigated using the Edinburgh Postnatal Depression Scale (EPDS) with a cutoff score of 14; the social support that they obtained was measured using the Social Support Questionnaire; and their coping styles were assessed using the Coping Styles Questionnaire.

**Results:**

Immediately after the earthquake, the incidence rate of depression in pregnant women was 35.2%, higher than that of the general pregnant population (7%-14%). The EPDS scores were significantly correlated with gestation age at the time of the earthquake, objective support, subjective support, use of support, negative coping style, and positive coping style. The regression analysis indicated that risk factors of prenatal depression include the number of children, relatives wounded, subjective support, and coping styles. A further analysis of the interaction between social support and two types of coping styles with depression showed that there was interaction effect between subjective social support and positive coping styles in relation to EPDS scores. There was an inverse relationship between low EPDS scores and positive coping styles and high social support, and vice versa.

**Conclusion:**

The timing of the occurrence of the earthquake may not necessarily affect the progress of the illness and recovery from depression, and psychological intervention could be conducted in the immediate aftermath after the earthquake. The impact of coping styles on prenatal depression appeared to be linked with social support. Helping pregnant women to adopt positive coping styles with good social support after a recent major earthquake, which is a stressor, may reduce their chances of developing prenatal depression.

## Introduction

On April 20, 2013, an earthquake measuring 7.0 on the Richter scale hit Lushan County of Ya’an City, which is located approximately 100 kilometers away from the city of Chengdu, in China’s Sichuan province. The earthquake resulted in 196 deaths, 11,470 injuries, and over 85 billion RMB in property damage [1]. Apart from economic losses, victims suffered from psychological trauma, especially depression [2–3]. Empirical studies have shown that major depression is common among earthquake victims [4–5]. Pregnant women who are experiencing great physical and psychological changes might be particularly vulnerable [6]. Studies on depression suffered by pregnant women after an earthquake indicated higher rates (13.1%-40.8%) [7–9] than among pregnant women in the general population (7%-14%) [10–12]. In addition, the women who lived in the epicenter immediately after a major earthquake may suffer greater depression. A study on public health conducted after the 2008 earthquake in Wenchuan found different physical and mental health outcomes depending on the extent to which local populations had been exposed to the earthquake [13]. A systematic review [14] of disasters and perinatal health also suggested that the extent to which pregnant women are exposed to a disaster, in which the time and place of the disaster are relevant factors, could pose different risks to their perinatal health. It is thought that the people living in the epicenter area shortly after the occurrence of an earthquake are under the greatest stress, and might be more prone to depression. Depression during pregnancy may have negative consequences for maternal and child health, with such consequences as increased rates of suicides, preterm births, and adverse psychological developments in the offspring [11, 15–17]. Therefore, it is necessary to investigate the depression of pregnant women and its risk factors at the epicenter immediately after a major earthquake in order to develop intervention strategies for this population. However, most research of prenatal depression after an earthquake focus on either the psychological impact to the pregnant women several months after the disaster [18], or to the target population living in a distance from the epicenter [19]. The depression of pregnant women and its risk factors at the epicenter immediately after an earthquake remains to be studied.

There were various studies that focused on the risk factors for depression [7, 11–12, 18–19]. Among those studies, social support has been regarded as a potential resource for relieving depression in pregnant women [20–22]. It was defined by Cooke [23] as a series of support accessible to an individual through social relations to other individuals, groups, and the larger community. Since Durkheim (1951) first established that social support has a positive effect on health, many researchers have found that it can serve as a mediator between stress and psychological problems [22, 24–26]. Thus, it is reasonable to assume that social support could also exert a similar function in helping pregnant women who have experienced a major earthquake, generally deemed to be a major stressful event. However, a number of researchers [27–30] suggested that the relationship between social support and psychological wellbeing might vary by culture and circumstances. For instance, emotion-focused support was more prevalent than instrumental support (e.g. money, transportation) in western culture, whereas the Asians such as Japanese exhibited the opposite pattern [27–31]. The link between perceived support and health under different cultural contexts might be an aspect of cross-national studies and should be prioritized in future studies. A study conducted by Murphy also found that social support might not always have a mediating effect on mental health [32]. Although two studies have indicated that there might be a correlation between social support and prenatal depression after an earthquake in Chinese population [7, 19], neither study provides sufficient information to justify that assumption. The cogency of the study conducted by Dong et al. [7], who investigated the mental health of pregnant women and their social support four years after the Sichuan earthquake of 2008, might have been weakened by the amount of time that had passed before the study was conducted. Although the study by Lau et al. [19] was conducted only 3 months after the 2008 earthquake in Wenchuan, the women were not from this epicenter but from Chengdu, which is 90 km away. In other words, the impact of social support on the depression of pregnant women at the epicenter immediately after a major earthquake remains unknown.

Apart from social support, effective coping strategies might also protect individuals from developing mental health problems when they experience an earthquake [33]. Lazarus and Richard [34] have defined coping as the effort that an individual makes to master demands that are appraised as exceeding or taxing his or her resources. In other words, how a pregnant woman appraises an event may shape its impact and how she responds. Thus, the outcomes of individuals under stress may also depend on their coping strategies. According to Folkman and Lazarus [35], coping strategies can be divided into the following eight styles: confrontive coping, distancing, self-control, seeking social support, accepting responsibility, escape-avoidance, planful problem-solving, and positive reappraisal. Stone and Neate suggested eight other coping strategies, such as distracting attention and relaxing [36]. The most frequently used categorization for coping styles were problem-focused and emotion-focused [37–38]. Problem-focused coping includes taking actions to resolve problems, as compared to emotion-focused coping, which aims at reducing feelings of distress related to stressful experiences. Some researchers categorized coping strategies into two other styles: approach or engagement coping, which focused on dealing with the stressor itself, and avoidance or disengagement coping, where people “cope” by not dealing with stressors [38]. Although the coping styles varied according to the researcher, they had some characteristics in common. For instance, seeking social support or engagement coping is behavior that is considered positive, while escape-avoidance is regarded as somewhat negative. The characteristics of positive and negative coping strategies were found to be easy to observe and understand in the real world. With this in mind, Xie designed a simplified questionnaire on coping styles that investigates coping styles according to positive or negative characteristics [39]. A number of studies have demonstrated the relationship between coping styles and depression and found that the avoidant or negative coping behaviours were associated with depression [38, 40–43]. Nevertheless, most of these studies focused on the general population or on non-disaster related stressors, leaving the association between coping styles and depression of pregnant women after a disaster largely unknown. In addition, it has been suggested that coping styles might affect the social support that is obtained, which would be further related to psychological wellbeing [44–46]. Rudnicki et al. [47] found that minority women who reported less social support satisfaction experienced greater avoidant coping styles, which would be associated with higher levels of depression. However, none of the studies explored the relationship between the social support and coping styles of pregnant women under a stressor such as an earthquake.

Since a number of studies focusing on the general population rather than on pregnant women after an earthquake have found that factors such as social support or coping styles are related to the onset and development of depression [24–26, 48], it can be assumed that depression, social support, and the coping styles of pregnant women after an earthquake may be interrelated. In addition, the literature has merely indicated the existence of a relationship between the depression of pregnant women and either social support or coping styles, but not both. The interrelationship between social support and coping styles after a disaster remains unclear. This lack of information hampers our efforts to explore those relationships and hence the potential to suggest effective interventions targeting either or both factors. Therefore, we conducted this study to examine the relationship between depression and the social support and coping styles of pregnant women in the aftermath of the Lushan earthquake. This study was conducted between 2 and 6 weeks after the earthquake, making it the first of its kind to study the depression of pregnant women, the social support that they obtained, and their coping styles immediately after a major earthquake. Since it is believed that the impact from an earthquake diminishes over time [14, 49], obtaining the data of women immediately after an earthquake can better indicate the impact of such a disaster. In addition, a number of studies have implied that the earlier that a psychological intervention can be implemented on victims after a major natural disaster, the better the results will be [48, 50–51]. Identifying risk and protective factors early after a disaster will facilitate the development of interventions. This study contributes by providing knowledge that is currently lacking, and discussing the implications for future research on interventions that can be implemented among such a population.

## Methods

### Study design

A cross-sectional survey was conducted on pregnant women from three hospitals in Ya’an City, which was the epicenter of the Lushan earthquake. Since the survey was conducted immediately after the disaster and challenges existed in finding the participants and delivering materials related to the investigation, the researchers did not have the time and resources to identify another group of women for comparison.

### Participants

One hundred and thirty pregnant women who experienced the earthquake in the epicenter area were recruited by convenience sampling when they came to the three hospitals for prenatal care. When the earthquake occurred, all major roads into Ya’an were blocked except to vehicles transporting disaster relief materials, and communication between people living in the epicenter area and those outside was affected. Therefore, it was quite difficult to deliver the related information materials to pregnant and postpartum women, and to recruit participants immediately after the earthquake. This made it impossible to obtain a large sample size and to carry out random sampling. On the basis of studies on depression in pregnant women after an earthquake [7–9], it was estimated that a sample of up to 190 would be appropriate [52]. However, as the number of deliveries at the three hospitals constituted over half of the total number of deliveries in Ya’an, this survey covered the majority of pregnant women who were accessible at the time.

Participants for this study needed to meet the following inclusion criteria: 1) those who had been pregnant for over 12 weeks when they experienced the Lushan earthquake; 2) who had lived in Ya’an during and after the earthquake; 3) who had no known history of auditory, language, or cognitive problems, and were able to communicate in Chinese.

In addition, both pregnant and postpartum women were targeted as participants, in accordance with our original study design. Nevertheless, considering the small number (only 15) of postpartum women that we were able to recruit for the study, due to our limited resources immediately after the earthquake, we did not analyze the results of the postpartum women.

### Procedures

From 2 to 6 weeks after the Lushan earthquake, three local volunteers who had been trained through telephone by three of the researchers (R.J, Y.J, & L.X) to work on the study instruments, interviewed the participants at the three hospitals in Ya’an. All of the participants were told the purpose of the study and gave their written informed consent to participate in the study before the questionnaires were administered to them. The interviews took place from May 4 to June 1, 2013. Data was collected only once and each instance of data collection lasted about 40 minutes. Code numbers, rather than names, appeared on the questionnaires. After all of the completed questionnaires were collected, two volunteers mailed 86 questionnaires and one volunteer carried 59 questionnaires back to Chengdu for data analysis. The data were entered and stored by one author (R.J) in a password-protected computer.

### Measures

The demographic data were collected through a self-designed questionnaire, which included socio-demographic data such as age, gestation age at the time of the earthquake, education, income, and injury and/or death of relatives. The main results of this study were obtained using three questionnaires: the Edinburgh Postnatal Depression Scale (EPDS), the Social Support Questionnaire-Chinese version (SSQ), and the Coping Styles Questionnaire (CSQ).

The EPDS is a self-rating scale with 10 items and was originally designed for assessing postpartum depression [53–54]. However, it had a similar effect on pregnant women, with desirable reliability and validity [19, 55]. For instance, in their study on pregnant women, Lau et al. [19] mentioned that the Cronbach’s Alpha coefficient for the EPDS was 0.76 and that the instrument demonstrated satisfactory concurrent validity with the Beck Depression Inventory and the General Health Questionnaire. The maximum score is 30, and a score of 14 or higher suggests a depressive illness of varying severity. Considering that all of the participants were Chinese, the Chinese version of the EPDS was used so that the participants would be better able to understand the questions [56–57].

The SSQ was designed by Xiao [58], and was proven to have acceptable psychometrics among Chinese university students [59] and farmers [60], with a test-retest reliability of 0.92, and good predictive validity. This questionnaire consists of three dimensions: objective support, subjective support, and usage of support. Objective support is visible or actual support. It includes material aids, and the existence of and participation in social networks. It also includes team relations, defined as the availability of stable marriages, and unsteady social relations such as informal organizations and temporal social interactions. Subjective support is a kind of support that is experienced or felt by oneself. It is defined as the experience of, and the degree of satisfaction felt in, being supported, understood, and respected. Usage of support is the third dimension of social support, and refers to the degree to which the available support is used [58].

The CSQ was revised by Xie [39] to suit the characteristics of Chinese people based on the Ways of Coping Questionnaire. The latter was designed by Folkman and Lazarus [61] and has two dimensions (positive coping and negative coping) with 20 items. Because the survey would be conducted among the Chinese population, and because after the earthquake conditions for conducting a survey were far from ideal, we used the simplified version of the questionnaire. This scale has acceptable psychometrics in studies on earthquake victims in China [62]. The test-retest reliability of the CSQ was 0.89 and the Cronbach’sαcoefficient was 0.90. The factor analysis indicated that the items could differentiate “negative” and “positive” factors and were in line with theoretical construct [62].

### Ethical considerations

The study was approved by the ethics committee of the West China Second University Hospital of Sichuan University. Written informed consent was obtained from the participants before the questionnaires were distributed to them.

### Data analysis

The cut-off score of the EPDS is 14, on the basis of which the incidence rate of depression was estimated. In a series of analyses of variance (ANOVA), the differences in the EPDS scores were compared by demographic factors. Possible confounders were: 1) demographic factors that are often used as confounders in other studies [63–65] focusing on disasters, e.g., age, income, relatives wounded and dead, etc., and 2) factors that have been identified in other studies [8–9, 19] as related to depression during pregnancy or postpartum depression (e.g., education, number of pregnancies and children, etc.). The Pearson’s correlation coefficient (r) was calculated to indicate the relationship among demographic data, EPDS scores, social support scores, and coping styles scores. In order to determine whether demographical factors, social support, and coping styles are significantly associated with the prenatal depression immediately after a major earthquake, a multiple linear regression analysis was conducted against the EPDS scores. The method of the regression analysis is stepwise, which involves entering the variables in blocks one by one rather than all at once. In addition, an interaction analysis was conducted to check the interaction between the main risk or protective factors, including social support and coping styles, on depression. All of the data were inputted using the software SPSS 20.0 for statistical analysis, and the statistical significance was set at p<0.05.

## Results

A total of 130 pregnant women were invited and agreed to participate in this survey, of whom 128 returned valid questionnaires, for a response rate of 98.5%.

### Demographic features and the incidence rate of depression during pregnancy

Forty-five of the 128 participants had EPDS scores of ≥14 (the EPDS cutoff point); thus the incidence rate of prenatal depression in this study was 35.2%. The incidence rates of prenatal depression in the various studies focusing on pregnant women after earthquakes in China are listed in Table 1. The chi-square test indicated that the incidence rate in the present study is similar to that of the other two studies [7, 9] (p = 0.255).

**Table 1:** Incidence rate of prenatal depression in different studies.

	Location of earthquake	N	N of depression	N of non-depression	Incidence rate
The present study	Lushan, China	128	45	83	35.2%
Dong et al. [3]	Wenchuan, China	252	87	165	34.5%
Qu et al. [5]	Wenchuan, China	311	127	184	40.8%
Pearson chi-square	2.730				
Sig. (2-sided)	0.255				

*P<.05 is statistically significant.

The mean EPDS score was 11.25±5.71. The mean scores for the objective, subjective, and support usage subscale were 8.48±2.79, 24.73±4.54, and 8.05±2.0, respectively. The mean scores for negative and positive coping styles were 24.69±7.23, 34.59±8.20, respectively.

The characteristics of the participants are outlined in Table 2. The mean age of the 128 participants was 26.91±5.46 years. The mean gestation age at the time of the earthquake was 33.19±7.84 weeks.

**Table 2:** Demographics, EPDS scores, social support, and coping styles of women who participated in the study (n = 128).

Demographic factors	Groups	N (%)
Age	≤20	16 (12.5%)
	21~30	77 (60.2%)
	≥31	35 (27.3%)
Marriage	Unmarried	8 (6.3%)
	Married	120 (93.8%)
Education	≤ Primary school	14 (10.9%)
	High school	66 (51.6%)
	Junior college	33 (25.8%)
	≥ Bachelor’s degree	15 (11.7%)
Occupation	Farming	60 (46.9%)
	Industrial worker	3 (2.3%)
	Service industry	12 (9.4%)
	Medicine	3 (2.3%)
	Public functionary	5 (3.9%)
	Business	8 (6.3%)
	Other	37(28.9%)
Family income	<5000	65 (50.8%)
	5000~9999	13 (10.2%)
	10000~19999	24 (18.8%)
	20000~49999	15 (11.7%)
	≥50000	11(8.6%)
Number of children	No children	61 (47.7%)
	One child	52 (40.6%)
	≥2 children	15 (11.7%)
Gestation age at the time of the earthquake	<28 weeks	23 (18%)
	≥28 weeks	105 (82%)
Wounded relatives	No	109 (85.2%)
	Yes	19 (14.8%)
Dead relatives	No	125 (97.7%)
	Yes	3 (2.3%)

### Multivariable analysis for EPDS scores against demographic data

The multivariable analysis was performed using one-way ANOVA to compare the EPDS scores for different demographic factors by group. EPDS scores were higher in women who were farmers (p = 0.001), had more than two children (p<0.001), were at or beyond their 28 week of gestation at the time of the earthquake (p<0.01), and/or had relatives injured (p<0.001) or killed (p = 0.001) as a result of the earthquake (See Table 3).

**Table 3:** ANOVA of EPDS scores against demographic data.

	Group	EPDS scores	F	Sig.
**Age**	≤20	10.19±6.19	0.891	0.413
	21~30	11.79±5.85		
	≥31	10.54±5.16		
**Marriage**	Unmarried	10.86±6.62	0.065	0.799
	Married	11.27±5.68		
**Education**	≤ Primary school	13.29±5.53	2.086	0.105
	High school	11.86±5.76		
	Junior college	10.21±5.89		
	≥ Bachelor’s degree	8.93±4.46		
**Occupation**	Farming	13.07±5.93	12.467	*0.001
	Others	9.65±5.03		
**Family income**	<5000	12.26±5.99	2.136	0.080
	5000~9999	13.0±7.05		
	10000~19999	9.29±4.98		
	20000~49999	10.00±3.91		
	≥50000	9.18±4.42		
**Number of Children**	No children	9.41±4.93	8.967	*0.000
	One child	12.19±5.99		
	≥2 children	15.47±4.87		
**Gestation age at the time of the earthquake**	<28 weeks	8.96±6.14	4.654	*0.033
	≥28 weeks	11.75±5.52		
**Wounded relatives**	No	10.41±5.38	21.544	*0.000
	Yes	16.30±5.40		
**Dead relatives**	No	11.00±5.53	11.033	*0.001
	Yes	21.67±2.51		

*P<.05 is statistically significant.

### Correlations among the scores for EPDS, social support, coping styles, and demographic data

EPDS scores were significantly correlated with gestation age at the time of the earthquake, with three dimensions of social support, and with two dimensions of coping styles (Table 4). Higher EPDS scores were significantly related with exposure to the earthquake later in pregnancy (r = 0.181, p<0.05). Moreover, lower objective support (r = -0.324, p<0.001), subjective support (r = -0.372, p<0.001), and support use (r = -0.320, p<0.001) were related to higher EPDS scores in the women. Likewise, higher EPDS scores were related to higher negative coping scores (r = 0.420, p<0.001) but lower positive coping scores (r = -0.328, p<0.001).

**Table 4:** Bivariate correlation analysis of scores in EPDS, social support, and coping styles, and of demographic data.

		EPDS	Age	Gestation age	Objective support	Subjective support	Support use	Negative coping	Positive coping
EPDS	r	1	-0.014	0.181*	-0.324**	-0.372**	-0.320**	0.420**	-0.328**
Age	r		1	-0.025	-0.045	0.001	-0.123	0.162	0.092
Gestation age	r			1	0.039	0.008	-0.002	0.026	-0.065
Objective support	r				1	0.375**	0.347**	-0.201*	0.141
Subjective support	r					1	0.379**	-0.300**	0.205*
Support use	r						1	-0.226*	0.137
Negative Coping	r							1	-0.089
Positive Coping	r								1

*Correlation is significant at 0.05 (2-tailed).

**Correlation is significant at 0.01 (2-tailed).

P<0.05 is statistically significant.

Although EPDS scores were significantly and inversely correlated with three dimensions of social support, and with two dimensions of coping styles, the correlation coefficients between them were moderate, with the value of r ranging from -0.32 to -0.42 (Table 4).

Apart from EPDS, coping style scores were correlated with certain dimension of social support. Higher negative coping style was related to lower objective support (r = -0.201, p = 0.023), lower subjective support (r = -0.300, p = 0.001), and lower support use (r = -0.226, p = 0.010). However, higher positive coping style was only significantly associated with higher subjective support (r = 0.205, p = 0.020) and higher EPDS scores (r = -0.328, p<0.001) (Table 4).

### Regression analysis against EPDS scores

In order to determine whether demographic factors, social support, and coping styles were significantly associated with prenatal depression immediately after a major earthquake, a multivariate linear regression analysis was further conducted against the EPDS scores. Since the number of dead relatives was less than 5, this variable was excluded. The results are shown in Table 5. The stepwise regression screened out five variables that could be put into the regression model. The five variables included number of children (β = 2.189), relatives wounded (β = 3.466), subjective support (β = -0.312), negative coping styles (β = 0.218), and positive coping styles (β = -0.134).

**Table 5:** Multivariate linear regression analysis against EPDS scores.

Model		R	R^2^	Unstandardized coefficients		Standardized coefficients	t	Sig.
				B	Std. Error	Beta		
1	(Constant)	0.420	0.177	3.062	1.641		1.866	0.064
	Negative coping			0.332	0.064	0.420	5.198	0.000
2	(Constant)	0.537	0.288	3.027	0.532		1.976	0.050
	Negative coping			0.300	0.060	0.380	4.996	0.000
	Wounded relatives			5.270	1.191	0.336	4.426	0.000
3	(Constant)	0.590	0.349	9.472	2.403		3.942	0.000
	Negative coping			0.286	0.058	0.362	4.942	0.000
	Wounded relatives			4.709	1.155	0.301	4.075	0.000
	Positive coping			-0.174	0.051	-0.249	-3.393	0.001
4	(Constant)	0.629	0.396	8.398	2.349		3.576	0.000
	Negative coping			0.275	0.056	0.348	4.912	0.000
	Wounded relatives			3.724	1.161	0.238	3.207	0.002
	Positive coping			-0.166	0.050	-0.238	-3.347	0.001
	Number of children			1.904	0.613	0.228	3.106	0.002
5	(Constant)	0.670	0.449	16.291	3.220		5.060	0.000
	Negative coping			0.218	0.056	0.276	3.885	0.000
	Wounded relatives			3.466	1.115	0.221	3.106	0.002
	Positive coping			-0.134	0.048	-0.193	-2.774	0.006
	Number of children			2.189	0.594	0.262	3.688	0.000
	Subjective support			-0.312	0.091	-0.249	-3.431	0.001

In the first step of the model, negative coping scores entered and accounted for 17.7% of the variance. Higher negative coping scores were associated with higher EPDS scores in the women. In the second step of the model, having a relative injured because of the earthquake entered into the model and accounted for an additional 11.1% of the EPDS scores. Women who had a relative injured had higher EPDS scores. In the third step, positive coping scores entered and explained another 6.1% of the EPDS scores. Lower positive coping scores were related to the women’s higher EPDS scores. Number of children entered into the model in the fourth step and accounted for 4.7% of the EPDS scores. The women with more than one child had higher EPDS scores than those with only one. In the final step, subjective support scores entered into the model and accounted for extra 5.3% of the EPDS scores. Altogether these five factors accounted for 44.9% of the EPDS scores.

### Interaction analysis of risk factors of depression

A correlation analysis (Table 4) showed that social support, coping styles, and EPDS scores were significantly correlated with each other. Although the multiple linear regression analysis found that not all types of social support could predict the variance of EPDS scores after the earthquake (Table 5), it is reasonable to investigate possible interactions between social support and coping styles on depression. In this connection, an interaction analysis was conducted. Since objective social support and support use were excluded from the regression model (Table 5), only subjective social support, which was an associated factor for depression, was used to create interaction terms with two coping styles.

**Fig 1:**
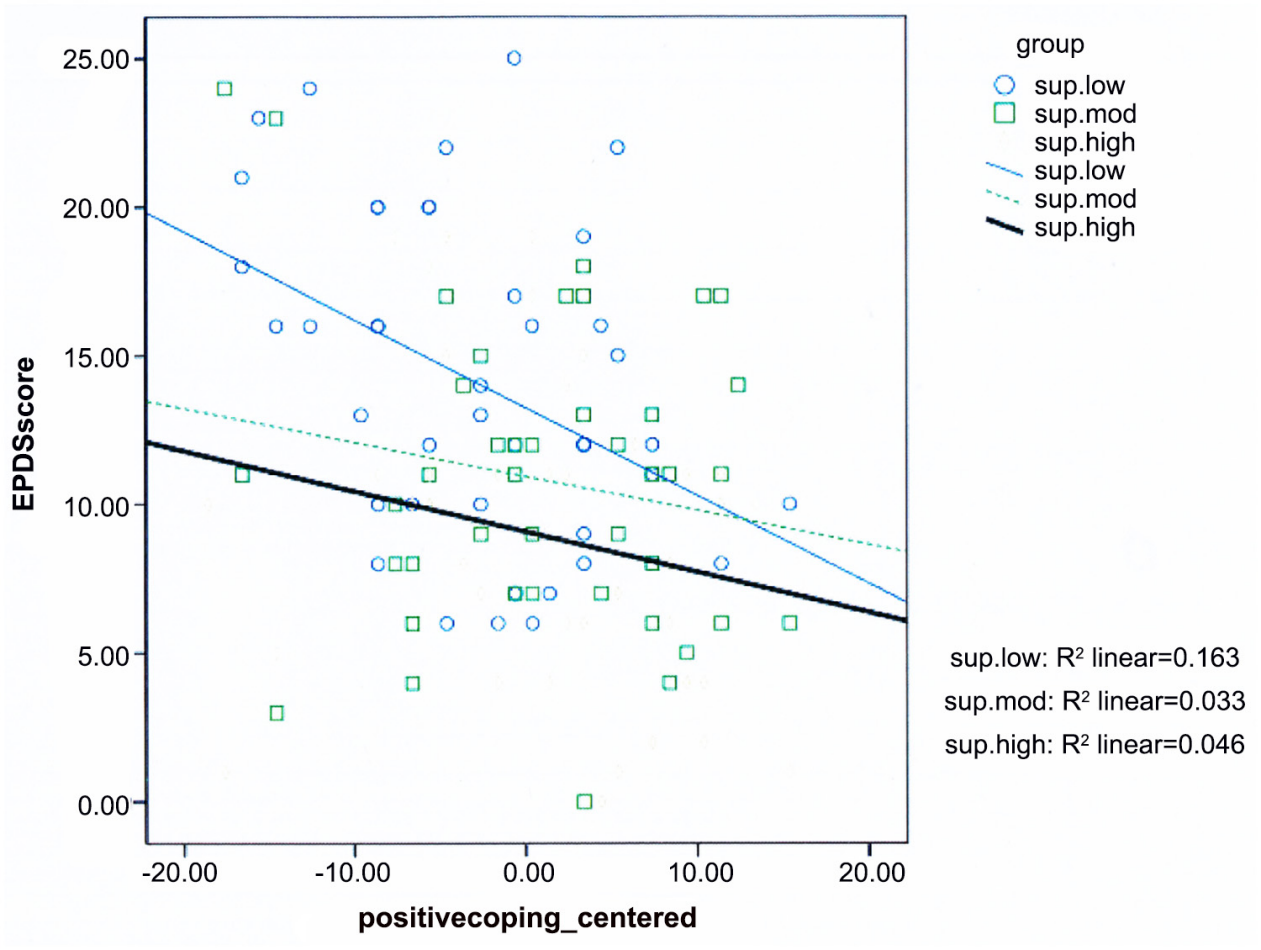
Effect of positive coping style on EPDS score as a function of subjective social support. * “sup.low” means low level of subjective social support. * “sup.mod” means moderate level of subjective social support.

The multiple linear regression analysis (Table 6) of subjective social support scores (centered), negative coping styles (centered), and positive coping styles (centered) in relation to the EPDS scores indicated that the interaction between subjective social support and positive coping styles (centered) had statistical significance. On this basis, a scatter diagram (Fig 1) for positive coping styles with different levels of subjective social support on EPDS scores was produced to illustrate the effects of these variables. Positive coping styles were found to be strongly associated with EPDS scores when subjective social support was high.

**Table 6:** Regression analysis of social support (centered), negative coping styles (centered), and positive coping styles (centered) in relation to EPDS scores.

Model		Unstandardized coefficients		Standardized coefficients	t	Sig.
		B	Std. Error	Beta		
1	(Constant)	11.230	0.448		25.058	0.000
	Support_centered	-0.262	0.099	-0.209	-2.641	0.009
	Negative coping_centered	0.278	0.062	0.352	4.511	0.000
	Positive coping_centered	-0.180	0.053	-0.259	-3.401	0.001
	Support_negativecoping_centered	0.013	0.013	0.078	1.043	0.299
	Support_positivecoping_centered	0.020	0.010	0.146	1.947	0.050

## Discussion

### The incidence rate of prenatal depression immediately after the earthquake

The rate of prenatal depression after the 2013 Lushan earthquake (35.2%) was similar to those results of the 2008 Wenchuan earthquake, which ranged from 34.5% to 40.8% [7, 9] (see Table 1). All these incidence rates were higher than those in the general population of pregnant women (7% to 14%) [10–12]. This implied that there might be somewhat a connection between higher rate of prenatal depression and being exposed to an earthquake. Although the timing of those studies differed, the similar higher rates of prenatal depression in different places after earthquakes indicated that the timing of these incidents might not necessarily affect the progress of such depression and its recovery. This suggests that psychological intervention could be conducted on pregnant women in the early stages following a major earthquake. Most researchers who have attempted to study interventions for earthquake victims have also suggested that interventions should be conducted as soon as possible after the disaster [48, 50–51].

### Pregnant women being at higher risk of developing prenatal depression following an earthquake

The results of a one-way ANOVA (Table 3) showed that the EPDS scores of pregnant women differed significantly depending on their occupation, number of children, gestation age at the time of the earthquake, and whether they had any wounded or dead relatives. Our analyses (Tables 4 and 5) showed that, five factors, namely, number of children, having wounded relatives, subjective social support, and coping styles (positive and negative) were significant determinants of prenatal depression immediately after the earthquake. The results of these analyses indicated that women who used higher levels of negative coping, lower levels of positive coping and who reported lower levels of subjective social support, particularly those who farmed, had more than two children, and had wounded relatives were at higher risk of prenatal depression following the earthquake.

The women, who worked in farming, had higher EPDS scores than those in other occupations. In mainland China, especially Sichuan, the major form of farming is terrace farming, which is generally considered particularly backbreaking and unremunerative work. Fewer and fewer people are choosing to stay in the countryside to farm [66–67]. The movement from rural to urban areas disproportionately involves the male population, leaving many females, elderly people, and children in the countryside [67]. As the main laborers in a family, women may assume more farming work after their husbands leave for cities, which may lead to a further deterioration in their psychological health [68]. Another possible reason for higher EPDS scores in farming women is that the delay in communication with their husbands might pose more uncertainty and stress on them. Most husbands of these women were away from their hometown for work, and it took some time before the women could contact their husbands and discuss family events with them. Therefore, following the earthquake, these women may not have an intimate person to share responsibility and provide support. As such, they would have had to make decisions for themselves and their families alone, which may make them feel burdened and stressed [68–70]. However, given the lack of an immediate comparison group, it was difficult to determine in the present study whether those women had higher EPDS scores before or after the earthquake. Nevertheless, it is clear that women whose occupation is farming need more psychological help immediately after the occurrence of a major earthquake.

It was also found from both multivariable analyses and regression analyses that the more children pregnant women had when the earthquake occurred, the higher their EPDS scores. This result may be attributed to their worries about the safety of young children. Young children are usually considered more vulnerable than adults in an earthquake because they are less capable of coping with emergency conditions in a disaster [71]. The findings from the study by Raposa et al. [72] suggest that increased levels of child-related stress could put mothers at risk of developing depression in the future. It is very likely that the worries about the safety of their younger children following the earthquake contributed to the occurrence of their depression. In addition, there was a higher incidence of depression among women with relatives who were wounded as a result of the earthquake. This finding is similar to those of other studies [8, 73]. According to the above analyses, the higher depression scores of mothers with more than one child or with relatives who were wounded might have been derived from the worries about the wellbeing of their families. Arranging better communication between pregnant women and their families immediately after the earthquake and let them know about their families’ well-being could help to alleviate some anxiety.

The EPDS scores were negatively associated with positive coping, and positively associated with negative coping (Table 4); and in the regression analysis the β of positive and negative coping styles were -0.134 and 0.218, respectively (Table 5). It would appear from this study that adopting positive coping styles has a positive impact and adopting negative styles has a negative impact on prenatal depression immediately after an earthquake. According to Lazarus and a number of researchers [34, 74–75], coping is a mediator between stress and health. When the earthquake occurred, the coping styles of women may play a role as a mediator in alleviating or deteriorating the impact of the stressor (earthquake) on health. The results of the current study are consistent with other findings in similar studies on coping during pregnancy. A systematic review suggested that higher depressive symptoms were associated with greater use of avoidance coping styles, which was similar in characterization with negative coping strategies assessed in our study, and low levels of problem-focused coping, which was similar to positive coping used in our study [38].

In the present study, our preliminary correlational analyses (Table 4) revealed that all the three types of social support were significantly associated with prenatal depression immediately after the earthquake. A number of studies found that social support is a risk/protective factor against the development of psychological problems during stress events [76–78]. The results of the present study further substantiate the function of this factor. However, not all of the three types of social support remained as statistically significant predictors of prenatal depression in the regression model. Only subjective social support was found to be a significant predictor of prenatal depression after an earthquake (Table 5). Subjective social support is the experience and degree of satisfaction felt in being supported, understood, and respected [58]. In contrast to objective social support, which represents the actual support that is obtained, subjective social support is more about the feeling of being supported. It is believed that subjective social support is the more important of the two kinds of support because a person’s psychological perception of reality could affect that individual’s behavior and development [58]. A number of researchers have demonstrated in their studies that the women who were more satisfied with support were less prone to depression [79–80]. Although objective social support was related to EPDS scores in the correlation analysis (Table 4), this factor was eventually excluded by the findings in the regression analysis (Table 5).

According to the above analyses, it could be concluded that using higher levels of negative coping, having lower levels of subjective social support, working as a farmer, having more than two children, and having wounded relatives were risk factors for prenatal depression following the earthquake. Regulating these factors immediately after an earthquake may decrease the depression of pregnant women and lead to favorable outcomes for those suffering from prenatal depression. Therefore, the following directions should be considered in the interventions to relieve the psychological problems of pregnant women: better attention and psychological support for farming women; more efficient communication between pregnant women and their families to let them knowing that their families were unhurt; higher quality medical services for wounded relatives; facilitating the feeling of being supported; and directing women to adopt more positive coping strategies and avoid negative strategies. For example, based on operational definitions for positive and negative coping styles [39], interventions could help women adopt actions associated with positive coping styles such as actively talking and listening, seeing events from a favorable perspective, and seeking solutions to problems, and avoid actions associated with negative coping styles such as evading reality, depending on others to solve problems, and performing bodily harm activities.

While the risk factors accounted for 44.9% of the variance in prenatal depression immediately after the earthquake (Table 5), there might be some other important risk factor/s that were not identified in the present study. According to Rich-Edwards et al. [81], history of depression is one of the strongest predictors of prenatal depression in women in general. History of depression may also explain a certain percentage of the variance in prenatal depression in the immediate aftermath of the earthquake. Furthermore, a systematic review on prenatal depression of women in general identified major life events and daily hassles as significant predictors of depression [22]. The occurrence of a major earthquake may cause greater life stress by disrupting daily life which may lead to depression [82–83]. Nevertheless, given that the current study had placed the focus solely on demographic factors, social support and coping styles, additional and possible risk factors cannot be fully understood in relation to prenatal depression during the aftermath of an earthquake. These factors may become the focus for future studies on prenatal depression immediately after earthquakes.

### Interaction analysis between social support and coping styles in relation to depression

The regression model (Table 6) and scatter diagram (Fig 1) for the interaction analysis indicated that the interaction term between subjective social support and positive coping styles reached statistical significance to less depression, but the term between social support and negative coping styles did not (Table 6). This result suggests that the effect of positive coping has on depression depended on the degree of subjective social support, so that there was no “unique” main-effect of positive coping. Coping capacities were different for women with varied subjective social support. It has been identified that higher subjective social support and positive coping styles, separately, are associated with lower tendency to experience prenatal depression in the current study (Table 5). However, the five factors including subjective social support and positive coping, could only account for 44.9% of the variance in depression. The emergence of interaction between subjective social support and positive coping may explain part of the variance that was not included in the 44.9%.

According to the interaction analysis, lower EPDS scores were observed when women, with lower subjective social support, adopted more positive coping strategies. This may be due to the situation that women with lower social support tend to adopt negative rather than positive coping strategies [47, 84]. In contrast to women with low social support, women who had high social support have invested more on positive coping styles. Analogous to the law of diminishing marginal effect [85], adopting more positive coping strategies may cause less increase of effect for women with high social support, and greater improvement of effect for those with low social support. This result imply that supportive relationships may impact on prenatal depression by helping women adapt to stress and changes in the aftermath of the earthquake. Clinical healthcare workers should pay more attention to other pregnant women who have negative coping styles and little social support. This also implies that a new intervention can be developed to prevent pregnant women from developing depression, involving by improving their feeling of being supported and helping them to develop positive coping styles in the aftermath of a major earthquake.

### The lower correlation coefficients between EPDS scores and social support and coping styles

It is worth mentioning that the correlation coefficients between EPDS scores and social support and coping styles appear to be moderate, with the value of r ranging from 0.32 to 0.42 (Table 4). According to Burnand [86], the strength of the association is moderate when r is > 0.30 and <0.45. It is difficult to explain this phenomenon, given the paucity of the relevant literature. The interaction of subjective social support and positive coping styles was found to have an inverse association with the EPDS scores (Fig 1). There might be some moderating effects between subjective social support and positive coping styles in that they provide essential encounter-actions immediately after a major earthquake to the development of depression during pregnancy. Future studies that target this aspect of the essential variables for deeper investigation are necessary.

## Limitations

Since the earthquake in this study caused great inconvenience to transportation, communications, and everyday life, the sample size was not very large and the participants were recruited by convenience sampling. It is also difficult to obtain a comparison group in the immediate aftermath of the disaster, which made us unable to compare the results of the prenatal women after the earthquake with their depression status before the earthquake or with a similar group that had not experienced the earthquake. These limitations may impact the interpretation of incidence rate of prenatal depression after the earthquake. However, the authors of this study managed to compare the incidence rate of depression of this study with the results of other study that focused on similar earthquakes in China. Furthermore, this study was performed using a cross-sectional approach under time and resource constraints in the immediate aftermath of an earthquake; thus, causality between variables could not be clearly demonstrated. Conducted only 2 to 6 weeks after the earthquake, it was only possible to collect information about the depression, social support, and coping styles of pregnant women. To obtain more rigorous and comparable evidence, a longer time frame for follow-up observations or an extended longitudinal study may be necessary.

## Conclusion

From this study, we were able to identify some risk factors that were associated with prenatal depression during the aftermath of earthquake in Ya’an. The occurrence of earthquake itself might somehow play a role to the incidence of depression during pregnancy. Although the timing of these incidents may not necessarily affect the progress of the depression and its recovery, there is a need to further investigate the relationship of earthquake and depression in pregnant women. Further interventions are needed for pregnant women immediately after an earthquake. There is a relationship between the utilization of positive coping styles and subjective social support and the severity of depression. By improving social support and helping pregnant women to positively cope with the aftermath of a major earthquake as a stressor, prenatal depression may be relieved.
